# Off-Label Use of Venetoclax in Patients With Acute Myeloid Leukemia: Single Center Experience and Data From Pharmacovigilance Database

**DOI:** 10.3389/fphar.2021.748766

**Published:** 2021-11-11

**Authors:** Lucia Gozzo, Calogero Vetro, Serena Brancati, Laura Longo, Daniela Cristina Vitale, Giovanni Luca Romano, Elisa Mauro, Paolo Fabio Fiumara, Cinzia Maugeri, Marina Silvia Parisi, Ilaria Dulcamare, Bruno Garibaldi, Andrea Duminuco, Giuseppe Alberto Palumbo, Francesco Di Raimondo, Filippo Drago

**Affiliations:** ^1^ Clinical Pharmacology Unit/Regional Pharmacovigilance Centre, A.O.U. Policlinico “G.Rodolico—S.Marco”, Catania, Italy; ^2^ Department of Biomedical and Biotechnological Sciences, University of Catania, Catania, Italy; ^3^ Haematology Unit, A.O.U. Policlinico “G.Rodolico—S.Marco”, Catania, Italy; ^4^ Postgraduate School of Hematology, University of Catania, Catania, Italy; ^5^ Department of Scienze Mediche Chirurgiche e Tecnologie Avanzate “G.F. Ingrassia”, University of Catania, Catania, Italy; ^6^ Department of Chirurgia Generale e Specialità Medico-Chirurgiche, University of Catania, Catania, Italy; ^7^ Centre for Research and Consultancy in HTA and Drug Regulatory Affairs (CERD) University of Catania, Catania, Italy

**Keywords:** venetoclax, acute myeloid leukemia, off-label, effectiveness, adverse event

## Abstract

The potent oral inhibitor of BCL2, venetoclax (VEN), used to treat adults with chronic lymphocytic leukaemia, has been approved in US for the treatment of naïve patients with acute myeloid leukemia (AML) unfit for intensive chemotherapy and recently in Europe, too. However, the drug has been used for years in combination with hypomethylating agents (HMAs) in patients not eligible to other treatment option, according to the so-called off-label use. We collected real-world data about patients treated with VEN + HMAs in the context of a pharmacovigilance project focused on the evaluation of the safety and effectiveness of drugs used for unapproved indication in Italian hospitals. From March to December 2020, 24 patients started treatment with VEN combined with HMAs. 21 patients have been assessed for response. Eleven (52%) patients reached complete remission (CR), and three patients (14%) CR with partial hematological recovery (CRh), with a median duration of response of 4.5 months (range 0.5–12.5). 19 patients experienced at least 1 adverse drug reaction (ADR), mostly serious, including 3 deaths (9% of ADRs; 12.5% of patients) in febrile neutropenia. Hematological toxicities and infections (cytopenia, neutropenia, febrile neutropenia, sepsis), were the most reported ADRs (84.4%). In general, neutropenic fever occurred more frequently in patients treated with decitabine (7 out of 9, 78%) compared to azacitidine (5 out of 15, 33%; *p* = 0.03), whereas response assessment did not differ based on used HMA (*p* = 0.1). These results confirm the benefit-risk profile of VEN in a real-world setting of patients with no adequate therapeutic options.

## Introduction

Venetoclax (VEN) is a potent oral inhibitor of the antiapoptotic molecule BCL2, used to treat adults with chronic lymphocytic leukaemia (CLL), in association with obinutuzumab in patients who have not previously been treated or with rituximab in patients who have received at least one previous treatment.

It is also used as monotherapy in patients with 17p deletion or TP53 mutation who cannot be treated with or have failed a B-cell receptor pathway inhibitor or in the absence of these genetic changes in adult patients who have failed both chemo-immunotherapy and a B-cell receptor pathway inhibitor.

Moreover, this drug has demonstrated activity and a good safety profile in patients with other hematological diseases, in particular acute myeloid leukemia (AML). The median age of AML patients at diagnosis is 68 years and this population has often limited effective treatment options due to ineligibility for intensive chemotherapy (Juliusson et al., 2009; National Cancer Institute, 2021). Less intense approaches, such as low-dose cytarabine (LDAC) or treatment with hypomethylating agents (azacitidine, decitabine), can be used but have been associated with poor response rates (complete remission, CR, plus CR with incomplete blood count recovery, CRi, rates less than 30%) and limited median survival times (< 6 months) (Kantarjian et al., 2012; Dombret et al., 2015; Amadori et al., 2016). This fact highlights the high unmet medical need for more effective and well-tolerated treatment options in this population, at least in part covered by the use of venetoclax.

Indeed, an extension of indication has been recently approved by the Food and Drug Administration (FDA) including the use in combination with azacitidine or decitabine or low-dose cytarabine for the treatment of naïve adult patients with newly-diagnosed AML who are unfit for intensive chemotherapy due to age or comorbidities.

This indication was initially granted under accelerated approval in November 2018, based on response rates (FDA, 2018). Efficacy was subsequently confirmed in two randomized, double-blind, placebo-controlled trials and FDA grants a regular approval in October 2020 (FDA, 2020).

In Europe this use was approved only in 2021, however the combination could have been prescribed in patients without other treatment options according to the national off-label regulation.

Therefore, since March 2020 it was possible in Italy to prescribe the combination of VEN + hypomethylating agents (HMAs) for adult patients with newly diagnosed AML not eligible for intensive induction chemotherapy, according to the Italian Law 648/96 (AIFA, 2020), which states that National Health System (NHS) can reimburse a drug 1) approved in other countries, or 2) currently used in clinical trials or 3) used for not approved indications (Law648, 1996).

Due to the fact that the benefit-risk profile of the drugs used in this setting could be not completely defined at the time of approval, a pharmacovigilance study has been approved in 2018 by the Italian Medicines Agency (AIFA) with the aim to monitor the use of drugs used in the context of the Italian Law 648/1996.

This study was conducted within this active pharmacovigilance project focused on the evaluation of the safety and effectiveness of drugs used for unapproved indication.

In particular, the aim of this analysis was to evaluate the safety and efficacy of VEN used in adult patients with newly diagnosed AML, according to Law 648/96.

## Materials and Methods

### Study Design

This prospective pharmacovigilance sub-study started on March 2020 at the University Hospital of Catania and enrolled AML patients, who met the eligibility criteria defined by the L.648/96, i.e., adult patients with newly diagnosed AML not eligible for intensive induction chemotherapy or aged 75 or more, were treated with HMAs combined with venetoclax.

### Treatment Regimens and Patients’ Response

Azacitidine was administered subcutaneously at a dose of 75 mg per square meter of body-surface area (BSA) from day 1–7. Decitabine was administered at a dose of 20 mg per square meter of BSA intravenously on days 1 through 5.

Venetoclax was administered orally, once daily, with food, starting the same day of the HMA with initial ramp-up, i.e., 100 mg on day 1, 200 mg on day 2 and from day 3 on the target dose of 400 mg was reached and continued until end of cycle. In all subsequent cycles, venetoclax was initiated at 400 mg, daily. To mitigate cytopenia and related clinical consequences, venetoclax was interrupted for recovery of blood counts. Dose modifications related to prophylactic anti-infective agents for venetoclax dose equivalency were implemented. Each cycle consisted of 28 days.

Response to treatment was assessed according to ELN response criteria (Döhner et al., 2017):

- complete remission (CR), defined as absolute neutrophil count (ANC) >1,000/μL, platelets >100,000/μL, red blood cell transfusion independence, and bone marrow with <5% blasts,
- CR with partial hematological recovery (CRh), defined as <5% of blasts in the bone marrow, no evidence of disease, and partial recovery of peripheral blood counts (platelets >50,000/μL and ANC >500/μL),
- morphologic leukemia-free state (MLFS), defined as bone marrow blasts <5%; absence of blasts with Auer rods; absence of extramedullary disease; no hematologic recovery required,
- partial response (PR), defined as decrease of bone marrow blast percentage to 5–25% and decrease of pretreatment bone marrow blast percentage by at least 50%,
- stable disease (SD), defined as absence of CR/CRi, PR, MLFS; and criteria for PD not met
- progressive disease (PD), defined as evidence for an increase in bone marrow blast percentage and/or increase of absolute blast counts in the blood.

### Data Collection

We collected data about baseline characteristics (age, AML type, performance status, citogenetic risk, and mutational status), treatment details [duration, concomitant medications, treatment modifications due to serious adverse drug reactions (ADRs), treatment discontinuation, reason for treatment discontinuation], with a specific focus on ADRs onset, and therapeutic response (including duration of remission and disease status at the last visit).

Each ADR report includes information about the patient, description of reactions (e.g., time to onset and recovery, seriousness, outcome, dechallenge, rechallenge, relevant laboratory tests), suspected and concomitant drugs (e.g., dosage, frequency and route of administration, therapeutic indication), clinical history and comorbidities. Suspected ADRs are grouped according to the Medical Dictionary for Regulatory Activities (MedDRA^®^) (Brown et al., 1999) and defined as serious if they were life-threatening or fatal, required hospitalization (or prolonged existing hospitalization), resulted in persistent or significant disability, or represented a congenital anomaly/birth defect or other medically important conditions (EMA, 2021). Moreover, these reports can be classified according to the results of causality assessment (definite, probable, possible, doubtful) performed according to the Naranjo Algorithm by pharmacovigilance expert (Naranjo et al., 1981).

Additionally, we conducted an analysis of ADR reports related to the use of venetoclax from the Italian spontaneous ADR reporting database (Italian National Network of Pharmacovigilance, Rete Nazionale di Farmacovigilanza, RNF). The RNF is a database that allows the collection, management and analysis of reports of all suspected ADRs in Italy. In particular, we analysed the Individual Case Safety Reports (ICSRs) collected into the RNF, summarized by type, source, sex, seriousness, outcome, and indication.

## Results

Starting from March to December 2020, a total of 24 eligible patients started treatment with venetoclax plus azacitidine (62.5%) or decitabine (37.5%; Tables 1, 2).

**TABLE 1:** Patient demographics and clinical characteristics at baseline.

Characteristic		%
Age, years, median (range)	73.5 (54–86)	—
AML type		
De novo	13	54%
Secondary to MDS or MDS/MPN	7	29%
Therapy-related	1	4%
Myeloid blast crisis from previous MPN	3	13%
ECOG performance status		
0	9	38%
1	8	33%
2	1	4%
3	5	21%
4	1	4%
Citogenetic risk		
Favorable	0	61%
Intermediate	15	
Poor	6	26%
Failed	3	13%
Mutations		
TP53	0	8%
FLT3-ITD	1 high ratio	
	1 low ratio	
IDH1/2	2 IDH1	8%
	2 IDH2 R140Q	8%
NPM1	0	

Abbreviations: AML, acute myeloid leukemia; ECOG, eastern cooperative oncology group; MDS, myelodysplastic syndrome; MPN, myeloproliferative neoplasm.

**TABLE 2:** Treatment details and disease status.

ID	Sex	Age	Treatment duration*	Concomitant medication	ADR	Treatment modification due to ADR	Treatment discontinuation	Reason for treatment discontinuation	Best treatment response	Duration of remission^§^	Subsequent treatments	Current disease status (last visit)
1	M	56	3	decitabine	X	x	X	Death	PR	—	—	PR
2	M	64	8	decitabine	X	—	X	Progression	CRh	24	venetoclax/azacitidine	Progression
3	F	74	12	decitabine	X	x	—	—	CR	50	—	CR
4	M	69	4	decitabine	x	X	X	Death	PR	—	—	Progression
5	F	76	5	decitabine	x	X	X	Progression	CR	14	Azacitidine alone	Relapse
6	M	80	9	azacitidine	x	—	—	—	CRh	41	—	CRh
7	F	73	0.3	azacitidine	x	x	X	Adverse event	NA	—	—	NA
8	M	86	3	azacitidine	x	—	X	Death	CRh	5	—	CRh
9	F	76	2	decitabine	x	x	X	Progression	CR	12	—	Progression
10	F	66	6	decitabine	x	x	X	Progression	CR	26	—	Relapse
11	F	79	5	azacitidine	—	—	X	Progression	PR	—	—	Progression
12	F	63	7	azacitidine	—	—	—	—	CR	28	—	CR
13	M	76	0	azacitidine	—	—	—	—	NA	—	—	NA
14	F	75	5	azacitidine	x	—	X	Adverse event	SD	—	—	SD
15	M	80	2	azacitidine	—	—	X	Progression	SD	—	—	Progression
16	M	71	5	azacitidine	x	x	—	—	CR	15	—	CR
17	M	80	4	azacitidine	x	x	—	—	CR	10	—	CR
18	M	76	3	azacitidine	—	—	—	—	CR	9	—	CR
19	F	70	3	decitabine	x	x	—	—	CR	8	—	CR
20	F	68	2	decitabine	x	x	—	—	CR	8	—	CR
21	M	54	1	azacitidine	x	x	X	Lost to follow-up	NA	—	—	NA
22	M	74	0	azacitidine	x	x	—	—	CR	5	—	CR
23	M	55	2	azacitidine	x	x	—	—	SD	—	—	SD
24	F	71	2	azacitidine	x	x	—	—	SD	—	—	SD

Abbreviations: ADR, adverse drug reaction; CR, complete remission; CRh, complete remission with incomplete hematologic recovery; PR, partial response; SD, stable disease; NA, not available; *months; §weeks.

Median age was 73.5 years (range 54–86). Patients were mainly of male sex (54.2%). The median follow-up was 3.3 months (range 0.5–14). Three patients out of 24 (13%) did not complete the first cycle of treatment. Two of them showed intolerance to the drug combination, manifesting as malaise and fatigue, arising the day after treatment start, leading to definitive treatment stop after 7 days, while one of them was lost-to-follow-up. Nine patients started treatment as in-patients, while remaining as out-patients. Dosage of venetoclax was reduced to 100 mg/day, after titulation, in three patients, cause concomitant treatment with posaconazole. Treatment with venetoclax has been temporally interrupted after cycle 1 in 4 patients out of 21 (19%), in order to let the hematological recovery, two of them reaching the CRh, 1 in PR and 1 with persistence of disease. In 3 cases, we observed a hematological recovery within 4 weeks, except the patient in PR, who showed a rapid progression of the disease. Remaining patients pursued to subsequent cycles directly at day + 29.

### Treatment Response and Adverse Events

21 patients (88%) have been assessed for treatment response. Among these patients, the best therapeutic response was CR in 11 (52%), whereas CRh was reached in three patients (14%), with a mean duration of response of 4.5 months (range 0.5–12.5). Three patients (14%) showed PR and 4 (19%) SD. Among the 14 patients showing CR or CRh, 3 (21%) relapsed after a median of 24 weeks and among the three patients with PR, 2 (66%) progressed 12 weeks after the reached PR, while 1 died due to infectious complications. Patients pursuing treatment received on average five cycles of treatment.

Up to date, 10 patients out of the evaluable 21 (48%) discontinued treatment permanently due to disease progression (*n* = 5/10; 50%), drug-related death due to febrile neutropenia (FN) (*n* = 2/10; 20%), FN in resistant patient (*n* = 1/10; 10%) or malaise (*n* = 2, 20%).

With regard to ADRs, 19 patients out of 24 (79%; 9 females and 10 males) experienced at least one ADR, and 10 of them more than ones (Table 3).

**TABLE 3:** Treatment-emergent adverse event.

ICSR ID	Patient ID	Suspected drugs	Sex/age	ADR	Seriousness	Outcome	Causality assessment
642796	1	venetoclax decitabine	M; 56	Febrile neutropenia, sepsis	Serious-death	Death	Possible
642799	1	venetoclax decitabine	M; 56	Neutropenia, inguinal abscess	Serious—hospitalization	Fully recovered	Possible
630562	2	venetoclax decitabine	M; 64	Prolonged cytopenia	Not serious	Not yet resolved	NA
645828	2	venetoclax azacitidine	M; 64	Febrile neutropenia	Serious—hospitalization	fully recovered	Possible
642794	3	venetoclax decitabine	F; 74	Neutropenia, dental abscess and tooth extraction	Serious—other clinically relevant condition	Fully recovered	Possible
644296	3	venetoclax decitabine	F; 74	Neutropenia	Serious—other clinically relevant condition	Fully recovered	Possible
678868	3	venetoclax decitabine	F; 75	Febrile neutropenia	Serious—other clinically relevant condition	fully recovered	Possible
644295	4	venetoclax decitabine	M; 69	Febrile neutropenia	Serious—hospitalization	Fully recovered	Probable
644297	4	venetoclax decitabine	M; 69	Febrile neutropenia	Serious-death	Death	Possible
644158	5	venetoclax decitabine	F; 76	Neutropenia	Serious—other clinically relevant condition	Fully recovered	Probable
644223	5	venetoclax decitabine	F; 76	Subdural hematoma	Serious—hospitalization	Improved	Probable
644280	5	venetoclax decitabine	F; 76	Febrile neutropenia	Serious—hospitalization	Fully recovered	Possible
640217	6	venetoclax azacitidine	M; 80	Melena	Serious—hospitalization	Not reported	Possible
678869	6	venetoclax azacitidine	M; 80	melena, pancytopenia, angiodysplasia	Serious—other clinically relevant condition	Not yet resolved	Possible
644294	7	venetoclax decitabine	F; 74	Abdominal pain, epigastralgia, dyspepsia, melena	Not serious	Not reported	NA
645829	8	venetoclax azacitidine	M; 86	Neutropenia, febrile neutropenia	Serious-death	Death	Possible
642337	9	venetoclax decitabina	F; 76	Febrile neutropenia, pancytopenia	Serious—hospitalization	Not yet resolved	Possible
643111	10	venetoclax decitabine	F; 67	Febrile neutropenia	Serious—other clinically relevant condition	Fully recovered	Probable
643113	10	venetoclax decitabine	F; 67	Neutropenia	Serious—other clinically relevant condition	Fully recovered	Probable
643114	10	venetoclax decitabine	F; 64	Neutropenia	Serious—other clinically relevant condition	Fully recovered	Possible
630889	14	venetoclax azacitidine	F; 75	Asthenia, hyperbilirubinemia, increased INR	Serious—hospitalization	Not yet resolved	Possible
678873	16	Venetoclax	M; 71	general malaise, neutropenia	Serious—other clinically relevant condition	improved	Possible
678872	17	venetoclax azacitidine	M; 80	Neutropenia	Serious—other clinically relevant condition	fully recovered	Possible
678709	19	venetoclax decitabine	F; 71	Neutropenia	Serious—other clinically relevant condition	improved	Possible
678707	20	venetoclax azacitidine	F; 68	Fever	Not serious	fully recovered	NA
678870	20	venetoclax azacitidine	F; 68	Neutropenia	Serious—other clinically relevant condition	Not reported	Possible
678706	21	venetoclax azacitidine	M; 54	Febrile neutropenia, Deterioration of general condition	Serious—hospitalization	Not reported	Possible
678874	22	venetoclax azacitidine	M; 74	Neutropenia	Not serious	Not reported	NA
681687	23	venetoclax azacitidine	M; 55	Febrile neutropenia	Serious—other clinically relevant condition	Fully recovered	Possible
681688	23	venetoclax	M; 55	pancytopenia	Serious—other clinically relevant condition	Fully recovered	Possible
678705	24	venetoclax azacitidine	F; 71	pancytopenia, hypocellular bone marrow	Serious—other clinically relevant condition	Not yet resolved	Possible
678708	24	venetoclax azacitidine	F; 71	Febrile neutropenia	Serious—other clinically relevant condition	improved	Possible

Abbreviations: ADR, adverse drug reaction; NA, not available.

Reported ADRs were mostly serious (*n* = 28, 87.5% of 32 total ADRs), including 3 deaths (9% of ADRs; 12.5% of patients) in FN, 1 in a patient with CRh at cycle 2 and 2 in patients in PR, 1 at cycle 2 and 1 at cycle 3. More than half of them showed a positive outcome (*n* = 4, 12.5% improved, and *n* = 15, 46.8% fully recovered). Hematological toxicities and infections, including cytopenia, neutropenia, FN and sepsis, were the most reported ADRs (84.4%). The causality assessment was available for all the serious ADRs with the following results: 23/28 (82%) “possible,” and 5/28 “probable” (17%).

Taking into account the first and subsequent cycles, FN occurred in 12 out of 24 patients (50%). Three of them (25%) were invasive fungal infection (IFI, 1 possible and 2 probable). Three patients died due to FN, 1 with resistant AML and 2 in CRh. All but three patients underwent to antifungal prophylaxis with fluconazole 400 mg/die. Only the concomitant treatment with posaconazole led to reduction of venetoclax dosage to 100 mg/die. No growth factors or antibiotic prophylaxis have been used. No differences were recorded between ADRs in in-patients and out-patients. The three FN-related deaths occurred at subsequent cycles as outpatients, two of them underwent the first cycle as in-patients and were dismissed with recovered blood count.

At first cycle of treatment, ADR occurred in 10 patients out of 24 (41%), 8 of them (33% of total cohort) had febrile neutropenia. As aforementioned, three patients did not complete the first cycle of treatment. At second and subsequent cycles, 19 patients were evaluable, since 2 out of the 21 patients that completed the first cycle did not pursue to the second cycle cause FN-related death and severe malaise. ADR occurred in 14 out of 19 patients (73%), 8 of them (42% of evaluable patients) had a febrile neutropenia, including the aforementioned three cases of IFI.

In general, neutropenic fever occurred more frequently in patients treated with decitabine (7 out of 9, 78%) compared to azacitidine (5 out of 15, 33%; *p* = 0.03). Response assessment did not differ based on used HMA (100 vs. 66% in DEC and AZA groups respectively, *p* = 0.1).

### Data From the Italian Spontaneous ADR Reporting Database

Data from RNF showed a total of 263 ICSRs in a 3-year period following VEN approval in Italy (August 2017-August 2020; Table 4; Supplementary Table S1). Almost all were spontaneous reporting (97%) from clinicians (93%). More than 85% were related to off-label use, in particular in patients with AML (*n* = 193; 73.4%). We found a high proportion (63.9%) of reports improperly including “off-label” as the unique ADR (Supplementary Table S2). Due to the lack of further information, we excluded these reports. The great majority was classified as serious (71.6%), in particular “other clinically relevant condition” (Supplementary Table S3). The reactions were fatal in more than 23% of cases (*n* = 22), due to disease progression in more than half of them (*n* = 13).

**TABLE 4:** Adverse drug reactions reported in Italy from venetoclax approval in 2017.

ICSR	n	%
Spontaneous	255	97
From non-interventional study	6	2.3
From individual use (compassionate use, named patient basis)	2	0.7
Total	263	100
Sex	n	%
F	94	35.7
M	166	63.1
—	3	1.1
Indication	N	%
*—*	9	3.4
ALL	2	0.8
AML	193	73.4
CLL	27	10.3
Lymphomas	21	8.0
Other	11	4.2

Abbreviations: ADR, adverse drug reaction; ALL, acute lymphatic leukemia; AML, acute myeloid leukemia; CLL, chronic lymphocytic leukaemia.

## Discussion

AML treatment still represents an important unmet medical need, in particular for older patients (aged > 60 years) and/or patients unfit for intensive chemotherapy (Appelbaum et al., 2006; Juliusson et al., 2009; Cortes and Mehta, 2021) as well as for those with relapsed or refractory disease (Rowe and Tallman, 2010; Ferrara et al., 2019) due to the lack of valuable therapeutic options, able to induce durable responses together with a good safety profile.

In recent years, the molecular characterization of the disease, that explains the heterogeneity of responses and relapses, led to the development of new therapeutic strategies for eligible patients (e.g., FLT3 inhibitors) (Lai et al., 2019; Perl, 2021). In patients receiving intensive chemotherapy for AML, the overexpression of anti-apoptotic BCL-2 family members has been associated with poor outcome (earlier relapse, reduced CR rates and overall survival-OS), (Del Poeta et al., 2003; Mehta et al., 2013). Thus, BCL2 inhibitors, such as venetoclax, might allow to overcome chemotherapy resistance by inducing apoptosis (Konopleva et al., 2006), as shown both by *in vitro* and *in vivo* studies (Souers et al., 2013).

Moreover, the association of HMAs, that reduce the level of anti-apoptotic proteins important for survival of AML cells other than BCL2, might block a potential VEN resistance pathway (Tsao et al., 2012). VEN combined with HMAs (AZA or DEC) in a dose-escalation study enrolling older AML patients (> 65 years) not eligible for intensive chemotherapy showed a high overall response rate (CR + CRh), irrespective of dosage, with a rapid time to response and a long duration of response. Common grade 3/4 ADRs reported in the study were hematological toxicities (neutropenia, febrile neutropenia, decreased white blood count, anemia, thrombocytopenia) and pneumonia (DiNardo et al., 2019).

These promising results led to FDA approval in 2018 for U.S.A. and to a widespread adoption of this regimen in other countries all over the world (Sekeres et al., 2020).

However, due to the lack of approval by regulatory agencies, in Europe the use of VEN in AML was off-label till May 2021. According to the European Medicines Agency (EMA), off-label use “relates to situations where the medicinal product is intentionally used for a medical purpose not in accordance with the authorised product information” (European Union, 2017). It is the practice of prescribing drugs for unapproved indications or age group, dose, or dosage frequencies, and/or route of administration other than those listed in the specific summary of product characteristics (SmPC). A major advantage of off-label use is the possibility to access to innovative treatments and the satisfaction of medical needs, especially in cases where no other options are available. Nevertheless, if the benefit-risk ratio is not completely defined, off-label prescription could increase the risk of inappropriate use, medical error and ADRs (Bellis et al., 2014). Therefore, this use should be carefully controlled and monitored, in order to ensure it occurs only in the presence of data supporting a favorable benefit-risk profile.

Currently, EU legislation does not regulate the way medicinal products are ultimately used in medical practice, including off-label prescribing, which is on the contrary regulated at national level (Gozzo et al., 2020).

For example, France introduced the Recommandations Temporaires d’Utilisation (RTU) in 2012 (Degrassat-Théas et al., 2015), a regulatory framework which enables the reimbursement of off-label drugs. Similarly, the Italian Law 648/1996 ensures a nationwide access to off-label drugs (but also drugs approved in other countries or currently used in clinical trials) included in specific lists updated according to criteria for appropriate use and monitoring defined in the light of clinical evidence (at least phase II trials) (Law648, 1996; Gozzo et al., 2020). All these drugs are included in specific lists updated based on new scientific evidence resulting from at least phase II clinical trials. The inclusion of a drug in these lists may be promoted for example, by clinicians, scientific societies, and patient associations which can submit a specific dossier reporting all the following information:

- the severity of the disease and absence of a valid therapeutic option;
- the rationale and clinical data (at least from Phase II studies) supporting the proposed treatment;
- the proposed therapeutic plan;
- the estimated number of patients who could benefit from treatment in Italy;
- the estimated expenditure for the proposed treatment (which will be reimbursed by the NHS);
- the list of ongoing clinical trials;
- the authorizative status of the medicinal products in Italy and abroad.

AIFA technical-scientific committee analyses the dossier and will approve the therapeutic use if a favorable benefit-risk profile is demonstrated. However, the value of the drug might not be confirmed by subsequent studies; in this case it will be excluded by the list and would no longer be prescribed.

Specifically, the use of venetoclax in patients with AML has been recognized in March 2020 within the legal framework of the Italian L. 648/1996 (AIFA, 2020) based on the results of phase II studies, subsequently confirmed by the VIALE-A trial (NCT02993523), a phase III, randomized (2:1), double-blind, placebo-controlled, multicenter trial which evaluated the efficacy and safety of VEN + AZA versus placebo + AZA (DiNardo et al., 2020). This trial showed a CR/CRh (64.7 vs. 22.8%, *p* < 0.001) and an OS advantage (14.7 vs. 9.6 months, hazard ratio for death, 0.66; 95% CI, 0.52 to 0.85; *p* < 0.001) for the VEN + AZA combination compared to AZA monotherapy in untreated patients who were ineligible for intensive chemotherapy. The median time to first response was 1.0 months with VEN + AZA combination and 2.6 months with AZA, and the duration of response was 17.8 and 13.9 months, respectively. All patients had at least 1 ADR, serious in 83% of the patients in the VEN + AZA group and 73% of those in the control group. The discontinuation of treatment between cycles due to adverse events occurred in 72% of the patients in the VEN + AZA group and 57% of the patients in the placebo + AZA group. Neutropenia and febrile neutropenia were more frequently observed in the VEN + AZA group compared to the control group (42 vs. 28% and 42 vs. 19%, respectively), as well as infections of any grade (84 vs. 67%).

Waiting for these confirmatory results, we collected data about all the AML patients treated in our centre with this regimen according to L. 648/1996, within a specific pharmacovigilance study. Although limited to few cases (with baseline characteristics comparable to those of the subjects enrolled in clinical trials), our results confirm the positive benefit-risk profile of VEN + HMAs in a real-world setting of patients with no adequate therapeutic options.

These results are in line with those of clinical trials, both in terms of efficacy and safety. In particular, we observed a high rate of composite complete remission (overall more than 60%), both rapid and durable in patients with longer follow-up. Due to the low number of patients, a subgroup analysis (eg., considering cytogenetic risk, mutational status, AML type) could not be carried out. The ADRs were expected, according to the known safety profile of the drugs (Seymour et al., 2016; Davids et al., 2018). Above all, the high percentage of serious neutropenia, FN and infections, including 9 hospitalizations/prolongation of hospitalization and 2 deaths in patients in CRh, confirms the need for an accurate monitoring of myelosuppression in this population (Jonas and Pollyea, 2019; Pratz et al., 2019; DiNardo et al., 2020; Gangat and Tefferi, 2020).

Looking at ADRs reported since 2017 (year of first commercialization of VEN in Italy), we found a higher proportion of reports related to VEN use in AML (46/95; 48%) compared to that of the approved use in CLL (27/95; 28%). Moreover, we observed a higher rate of reporting “disease progression” in AML (24/46, 52%) compared to CLL (“lack of response” in 2/27, 7%). This difference may be secondary to an increased attention given to the therapeutic response to an off-label drug, as well as the under-reporting of expected adverse events common to all the therapeutic uses.

The main limitation of this work is undoubtedly the small number of patients enrolled, being a single centre study. Moreover, under-reporting and missing information are well known limitations of the pharmacovigilance reporting system. However, the strength of this study is the description of the use of VEN in a real-world setting as soon as the drug has been available in Italy in this population.

## Conclusion

In conclusion, the off-label prescription according to L. 648/96 of the combination VEN plus HMA allowed to treat unfit AML patients with a good treatment option, demonstrating a high and durable response rate. The frequent onset of febrile neutropenia requires special considerations on management of these patients. The strict monitoring of off-label drugs represents a valuable way to rapidly collect real-world evidence supporting an appropriate use in clinical practice.

## Data Availability

The raw data supporting the conclusion of this article will be made available by the authors, without undue reservation.
