# Supplementary material for: Off-Label Use of Venetoclax in Patients With Acute Myeloid Leukemia: Single Center Experience and Data From Pharmacovigilance Database
Source: Front Pharmacol. 2021 Nov 11;12:748766. doi: 10.3389/fphar.2021.748766 (PMC8660092; doi:10.3389/fphar.2021.748766)
Supplement: Supplementary file 1 [file DataSheet1.docx]

**Supplementary table 1:** Individual Case Safety Reports (ICSRs) related to the use of venetoclax from the Italian spontaneous ADR reporting database (Italian National Network of Pharmacovigilance, Rete Nazionale di Farmacovigilanza, RNF) in a 3-year period following venetoclax approval in Italy (August 2017-August 2020).

| **Type** | **ICSR ID** | **Date** | **Age** | **Sex** | **Seriousness** | **Outcome** | **Source** | **ADR** | **Indication** |
| --- | --- | --- | --- | --- | --- | --- | --- | --- | --- |
| Spontaneous | 454595 | 19/01/2018 | 47 | F | Not serious | Fully recovered | MD | Diarrhea | Chronic lymphocytic leukemia |
| Spontaneous | 464773 | / | / | M | Not serious | Not available | MD | Off-label use | Chloroma - myeloid sarcoma |
| Spontaneous | 469009 | 30/03/2018 | 73 | M | Not serious | Not available | MD | Off-label use | Refractory cancer |
| Spontaneous | 469010 | 23/03/2018 | 21 | M | Not serious | Not available | MD | Off-label use | Refractory cancer |
| Spontaneous | 471687 | / | 50 | F | Not serious | Not available | MD | Off-label use | Acute myeloid leukemia |
| Spontaneous | 471689 | / | / | M | Not serious | Not available | MD | Off-label use | / |
| Spontaneous | 472405 | 10/04/2018 | 52 | F | Serious—hospitalization | Fully recovered | MD | Increased transaminase | Chronic lymphocytic leukemia |
| Spontaneous | 472879 | / | / | F | Not serious | Not available | MD | Off-label use, disease progression | Acute myeloid leukemia |
| Spontaneous | 475459 | / | 62 | M | Not serious | Not available | MD | Off-label use | Acute myeloid leukemia |
| Spontaneous | 478051 | / | / | M | Serious-death | Death | MD | Disease progression | / |
| From non-intervantional study | 478875 | / | / | M | Not serious | Not available | Healthcare professional (other) | Drug resistance | Chronic lymphocytic leukemia |
| From non-intervantional study | 479001 | / | / | F | Not serious | Not available | Healthcare professional (other) | Lack of therapeutic response | Chronic lymphocytic leukemia |
| Spontaneous | 479366 | 01/05/2018 | 51 | M | Not serious | Not available | MD | Off-label use | Acute myeloid leukemia |
| Spontaneous | 479660 | 21/05/2018 | 46 | F | Not serious | Not available | MD | Off-label use | Acute myeloid leukemia |
| Spontaneous | 479896 | 01/05/2018 | / | M | Not serious | Not available | MD | Off-label use | Acute myeloid leukemia |
| Spontaneous | 480104 | / | 65 | M | Serious—other clinically relevant condition | Not available | MD | Off-label use | Acute myeloid leukemia |
| Spontaneous | 481118 | 01/01/2018 | 55 | M | Not serious | Not available | MD | Off-label use | Acute myeloid leukemia |
| Spontaneous | 481216 | 01/05/2018 | 50 | F | Not serious | Not available | MD | Off-label use | Acute myeloid leukemia |
| Spontaneous | 483819 | 01/06/2018 | 25 | M | Not serious | Not available | MD | Off-label use | Acute myeloid leukemia |
| Spontaneous | 485778 | / | / | M | Not serious | Not yet resolved | MD | Off-label use | Acute myeloid leukemia |
| Spontaneous | 486121 | 01/01/2018 | 50 | F | Serious—other clinically relevant condition | Fully recovered | MD | Kidney failure, Seizure, Tumor lysis syndrome, Hypercreatinemia | Acute myeloid leukemia relapsed |
| Spontaneous | 486122 | 01/01/2018 | 62 | M | Serious-death | Death | MD | Disease progression | Acute myeloid leukemia |
| Spontaneous | 486618 | 04/06/2018 | 60 | F | Not serious | Not available | MD | Off-label use | Acute myeloid leukemia |
| Spontaneous | 487226 | 28/05/2018 | 54 | F | Serious—hospitalization | Fully recovered | MD | Tumor lysis syndrome | Acute myeloid leukemia relapsed |
| Spontaneous | 489056 | 18/06/2018 | 50 | F | Not serious | Not yet resolved | MD | Off-label use | Acute myeloid leukemia |
| Spontaneous | 489474 | 11/06/2018 | 57 | M | Not serious | Not available | MD | Off-label use | Acute myeloid leukemia |
| Spontaneous | 489475 | / | 68 | M | Not serious | Not available | MD | Off-label use | Acute myeloid leukemia |
| Spontaneous | 489476 | 01/01/2018 | 60 | F | Serious-death | Death | MD | Disease progression | Acute myeloid leukemia |
| Spontaneous | 489477 | 04/06/2018 | 51 | M | Not serious | Not available | MD | Off-label use | Acute myeloid leukemia |
| Spontaneous | 490731 | 02/07/2018 | 53 | F | Not serious | Fully recovered | MD | Increased transaminase | Chronic lymphocytic leukemia |
| From non-intervantional study | 490783 | 21/07/2018 | 70 | F | Serious—other clinically relevant condition | Improved | MD | Neutropenia | Chronic lymphocytic leukemia |
| Spontaneous | 491889 | 20/07/2018 | 70 | F | Serious-death | Death | MD | Disease progression | Acute myeloid leukemia |
| Spontaneous | 491890 | 23/07/2018 | 73 | M | Not serious | Not yet resolved | MD | Off-label use | Acute myeloid leukemia |
| Spontaneous | 494747 | 04/08/2018 | 54 | M | Not serious | Improved | Healthcare professional (other) | Itching, Neck rash, Eyelid rash | / |
| Spontaneous | 496618 | / | / | M | Serious-death | Death | MD | Off-label use | Acute myeloid leukemia |
| Spontaneous | 497597 | / | 41 | F | Not serious | Not available | MD | Off-label use | Acute myeloid leukemia |
| Spontaneous | 497599 | / | 71 | M | Not serious | Not available | MD | Off-label use | Mantle cell lymphoma |
| Spontaneous | 497792 | / | 50 | F | Not serious | Not available | MD | Off-label use | Chloroma - myeloid sarcoma |
| Spontaneous | 497793 | / | 67 | M | Not serious | Not yet resolved | MD | Off-label use | Non-Hodgkin's lymphoma |
| Spontaneous | 497794 | 01/08/2018 | 68 | M | Not serious | Not available | MD | Off-label use | Acute myeloid leukemia |
| Spontaneous | 497795 | 01/08/2018 | 77 | M | Not serious | Not available | MD | Off-label use | Acute myeloid leukemia |
| Spontaneous | 497798 | / | 60 | M | Serious—other clinically relevant condition | Not available | MD | Disease progression | Acute myeloid leukemia relapsed |
| Spontaneous | 497944 | / | 50 | F | Not serious | Not available | MD | Off-label use | Acute myeloid leukemia relapsed |
| Spontaneous | 497945 | / | 61 | F | Not serious | Not available | MD | Off-label use | Acute myeloid leukemia |
| Spontaneous | 499074 | / | 56 | F | Not serious | Not available | MD | Off-label use | Acute myeloid leukemia |
| Spontaneous | 501467 | 01/09/2018 | 47 | F | Not serious | Not available | MD | Off-label use | Acute myeloid leukemia |
| Spontaneous | 501982 | 01/01/2018 | 64 | F | Serious-death | Death | MD | Disease progression | Acute myeloid leukemia |
| Spontaneous | 502321 | / | 67 | M | Not serious | Not available | MD | Off-label use | Acute myeloid leukemia |
| Spontaneous | 502474 | 01/09/2018 | 70 | F | Not serious | Not available | MD | Lack of therapeutic response | Mantle cell lymphoma |
| Spontaneous | 503004 | / | / | F | Serious-death | Death | MD | Off-label use | Acute myeloid leukemia |
| Spontaneous | 503008 | 01/01/2018 | 60 | F | Not serious | Not available | MD | Off-label use | Acute myeloid leukemia |
| Spontaneous | 503905 | / | 47 | F | Serious-death | Death | MD | Disease progression | Acute myeloid leukemia |
| Spontaneous | 504301 | / | 75 | M | Serious—other clinically relevant condition | Not yet resolved | MD | Off-label use | Mantle cell lymphoma |
| Spontaneous | 505211 | / | 62 | F | Not serious | Fully recovered | MD | Off-label use, pulmunary infection | Acute myeloid leukemia |
| Spontaneous | 505359 | 09/10/2018 | 78 | M | Not serious | Not available | MD | Off-label use | Acute myeloid leukemia |
| Spontaneous | 505360 | 08/10/2018 | 69 | F | Not serious | Not yet resolved | MD | Off-label use | Acute myeloid leukemia |
| Spontaneous | 505427 | / | 46 | M | Serious—other clinically relevant condition | Fully recovered | MD | Disease progression | Acute myeloid leukemia |
| Spontaneous | 505575 | 01/06/2018 | 25 | M | Serious-death | Death | MD | Disease progression | Acute myeloid leukemia |
| Spontaneous | 505934 | / | 50 | F | Serious-death | Death | MD | Disease progression | Chloroma - myeloid sarcoma |
| Spontaneous | 505973 | 28/08/2018 | 67 | M | Serious-death | Death | MD | Off-label use | Diffuse large B-cell lymphoma |
| Spontaneous | 506512 | 04/10/2018 | 55 | M | Not serious | Not available | MD | Off-label use | Acute myeloid leukemia |
| Spontaneous | 506513 | 08/10/2018 | 70 | M | Not serious | Not available | MD | Off-label use | Acute myeloid leukemia |
| Spontaneous | 506741 | 01/10/2018 | / | M | Not serious | Not available | MD | Off-label use | Acute myeloid leukemia |
| Spontaneous | 506744 | 01/08/2018 | 26 | F | Not serious | Not available | MD | Off-label use | Acute myeloid leukemia |
| Spontaneous | 506745 | 01/09/2018 | 57 | M | Not serious | Not available | MD | Off-label use | Acute myeloid leukemia |
| Spontaneous | 506855 | 01/10/2018 | 75 | M | Not serious | Not available | MD | Off-label use | Acute myeloid leukemia |
| Spontaneous | 507080 | 01/09/2018 | 70 | M | Not serious | Not available | MD | Off-label use | Acute myeloid leukemia |
| Spontaneous | 507082 | / | 76 | M | Serious—other clinically relevant condition | Not available | MD | Off-label use, disease progression | Acute myeloid leukemia |
| Spontaneous | 507083 | / | 70 | F | Serious—other clinically relevant condition | Not available | MD | Disease progression | Acute myeloid leukemia |
| Spontaneous | 507084 | 09/10/2018 | 78 | M | Not serious | Not available | MD | Off-label use | Acute myeloid leukemia |
| Spontaneous | 507085 | 12/10/2018 | 75 | M | Not serious | Not available | Healthcare professional (other) | Off-label use | Mantle cell lymphoma relapsed |
| Spontaneous | 507230 | 01/01/2018 | 40 | M | Serious-death | Death | MD | Disease progression | Acute myeloid leukemia |
| Spontaneous | 507486 | / | 78 | M | Serious-death | Death | MD | Off-label use | Acute myeloid leukemia |
| Spontaneous | 507488 | / | 50 | F | Serious-death | Death | MD | Disease progression | Acute myeloid leukemia |
| Spontaneous | 507861 | 15/10/2018 | / | M | Not serious | Not available | MD | Off-label use | Acute myeloid leukemia |
| Spontaneous | 507924 | 15/10/2018 | / | M | Not serious | Not available | MD | Off-label use | Acute myeloid leukemia |
| Spontaneous | 509626 | 25/10/2018 | 69 | M | Not serious | Not available | MD | Off-label use | Acute myeloid leukemia |
| Spontaneous | 510316 | 30/10/2018 | 64 | M | Serious—hospitalization | Improved | MD | Anemia, Asthenia, Increased Bilirubin, dyspnea, High Reticulocyte Count, Increased LDH, Positive Direct Coombs Test, Positive Indirect Coombs Test | Chronic lymphocytic leukemia |
| Spontaneous | 511434 | / | 22 | M | Not serious | Not available | MD | Off-label use | Acute myeloid leukemia |
| Spontaneous | 511555 | / | / | M | Not serious | Not available | MD | Off-label use | Acute myeloid leukemia refractory |
| Spontaneous | 511556 | 30/10/2018 | 18 | M | Not serious | Not yet resolved | MD | Off-label use | Acute myeloid leukemia |
| Spontaneous | 511715 | / | / | M | Serious—other clinically relevant condition | Not available | MD | Autoimmune hemolytic anemia | / |
| Spontaneous | 511792 | / | / | M | Not serious | Not yet resolved | MD | Off-label use | Acute myeloid leukemia |
| Spontaneous | 511793 | / | / | M | Not serious | Not available | MD | Off-label use | Acute myeloid leukemia |
| Spontaneous | 513027 | / | / | M | Not serious | Not available | MD | Off-label use | Acute myeloid leukemia |
| Spontaneous | 513255 | / | / | F | Not serious | Not available | MD | Off-label use | Mantle cell lymphoma |
| Spontaneous | 514148 | / | 28 | M | Serious—other clinically relevant condition | Not available | MD | Off-label use, Hematopoietic stem-cell transplantation (HSCT) | Acute myeloid leukemia refractory |
| Spontaneous | 514795 | 21/11/2018 | / | M | Not serious | Not available | MD | Off-label use | Multiple myeloma |
| Spontaneous | 515399 | / | / | M | Not serious | Not available | MD | Off-label use | Acute myeloid leukemia |
| Spontaneous | 515430 | 21/11/2018 | 68 | F | Serious—hospitalization | Not available | MD | Dyspnea, Orthopnea, Pleural effusion, Tachycardia, Ventricular dysfunction | Chronic lymphocytic leukemia |
| Spontaneous | 515643 | / | / | F | Not serious | Not available | MD | Off-label use | Acute myeloid leukemia |
| Spontaneous | 515644 | / | / | M | Not serious | Not available | MD | Off-label use | Acute myeloid leukemia |
| Spontaneous | 515645 | / | 75 | F | Not serious | Not available | MD | Off-label use | Mantle cell lymphoma |
| Spontaneous | 515735 | / | / | F | Not serious | Not available | MD | Off-label use | Waldenstrom macroglobulinemia |
| Spontaneous | 515736 | 13/11/2018 | / | M | Not serious | Not available | MD | Off-label use | Acute myeloid leukemia |
| Spontaneous | 516190 | / | / | M | Not serious | Not available | MD | Off-label use | Acute myeloid leukemia refractory |
| Spontaneous | 517640 | 01/01/2018 | 38 | M | Serious-death | Death | MD | Off-label use | Mantle cell lymphoma |
| Spontaneous | 518203 | / | 69 | M | Serious—other clinically relevant condition | Fully recovered | MD | Sepsis, Deterioration of general health status | Acute myeloid leukemia |
| Spontaneous | 519902 | 29/11/2018 | 42 | M | Not serious | Not available | MD | Off-label use | Acute myeloid leukemia |
| Spontaneous | 520817 | / | / | F | Serious—other clinically relevant condition | Not available | Healthcare professional (other) | Disease progression | Diffuse large B-cell lymphoma |
| Spontaneous | 520901 | 01/09/2018 | 57 | M | Serious—other clinically relevant condition | Fully recovered | MD | Disease progression | Acute myeloid leukemia |
| Spontaneous | 520902 | / | 55 | M | Not serious | Not available | MD | Lack of therapeutic response | Acute lymphoblastic leukemia |
| Spontaneous | 520903 | / | 69 | F | Not serious | Not available | MD | Off-label use | Acute myeloid leukemia refractory |
| Spontaneous | 524194 | / | 35 | M | Not serious | Not available | MD | Off-label use | Acute myeloid leukemia refractory |
| Spontaneous | 524726 | 24/10/2018 | 63 | F | Serious-death | Death | MD | Off-label use, infection | Acute myeloid leukemia |
| Spontaneous | 524727 | 01/01/2018 | 69 | F | Serious-death | Death | MD | Off-label use, infection | Acute myeloid leukemia |
| Spontaneous | 524982 | 09/01/2019 | / | M | Not serious | Not available | MD | Off-label use | Acute myeloid leukemia |
| Spontaneous | 525326 | 01/01/2019 | 46 | M | Serious-death | Death | MD | Neutropenic sepsis, bacterial sepsis | Acute myeloid leukemia |
| Spontaneous | 525688 | / | 49 | F | Not serious | Not available | MD | Off-label use | Acute myeloid leukemia refractory |
| Spontaneous | 526914 | 01/12/2018 | / | M | Serious-death | Death | MD | Septic Shock | Acute myeloid leukemia |
| Spontaneous | 526917 | / | / | M | Serious-death | Death | MD | Septic Shock | Acute myeloid leukemia |
| Spontaneous | 527379 | 18/01/2019 | 30 | M | Serious—hospitalization | Fully recovered | MD | Febrile Neutropenia, weight loss, disease progression | Peripheral T-cell lymphoma |
| Spontaneous | 527644 | / | 71 | M | Serious—other clinically relevant condition | Not available | MD | Disease progression | Acute myeloid leukemia |
| Spontaneous | 527756 | / | 64 | F | Serious-death | Death | MD | Disease progression | Acute myeloid leukemia |
| Spontaneous | 527957 | / | 75 | F | Not serious | Not available | MD | Off-label use | Acute myeloid leukemia refractory |
| Spontaneous | 528242 | 24/01/2019 | / | M | Not serious | Not yet resolved | MD | Off-label use | Acute myeloid leukemia |
| Spontaneous | 528888 | / | / | M | Serious-death | Death | MD | Disease progression | Acute myeloid leukemia |
| Spontaneous | 529238 | 25/01/2019 | / | M | Not serious | Not available | MD | Off-label use | Acute myeloid leukemia |
| Spontaneous | 529389 | / | 62 | M | Not serious | Not available | MD | Off-label use | Mantle cell lymphoma |
| Spontaneous | 530086 | 01/12/2018 | 33 | M | Not serious | Not available | MD | Off-label use | Acute myeloid leukemia |
| Spontaneous | 531487 | / | 35 | F | Not serious | Not available | MD | Off-label use | Acute myeloid leukemia |
| Spontaneous | 531820 | / | / | F | Not serious | Not available | MD | Off-label use | Acute myeloid leukemia |
| Spontaneous | 532520 | / | 72 | F | Not serious | Not available | MD | Off-label use | Acute myeloid leukemia |
| Spontaneous | 532521 | / | 46 | M | Not serious | Not available | MD | Off-label use | Acute myeloid leukemia |
| Spontaneous | 532647 | / | 70 | M | Serious—other clinically relevant condition | Not available | MD | Off-label use | Acute myeloid leukemia refractory |
| Spontaneous | 532850 | / | 77 | M | Serious—other clinically relevant condition | Not available | MD | Off-label use | Acute myeloid leukemia refractory |
| Spontaneous | 533718 | / | / | M | Serious—hospitalization | Not available | MD | Abnormal blood potassium | / |
| Spontaneous | 534090 | 25/02/2019 | 77 | F | Not serious | Not available | MD | Off-label use, Xerotic eczema, Itching | Mantle cell lymphoma refractory |
| Spontaneous | 534157 | / | 42 | F | Not serious | Not available | MD | Off-label use | Acute myeloid leukemia refractory |
| Spontaneous | 534159 | / | 65 | M | Serious—other clinically relevant condition | Not available | MD | Off-label use | Acute myeloid leukemia refractory |
| Spontaneous | 535713 | / | / | M | Not serious | Not available | Pharmacist | Hypercalcaemia | Chronic lymphocytic leukemia |
| Spontaneous | 536102 | 28/02/2019 | / | F | Not serious | Not available | MD | Off-label use | Mantle cell lymphoma |
| Spontaneous | 537049 | / | 73 | F | Not serious | Not available | MD | Off-label use | Acute myeloid leukemia refractory |
| Spontaneous | 537053 | / | 61 | M | Not serious | Not available | MD | Off-label use | Acute myeloid leukemia |
| Spontaneous | 538166 | / | 81 | F | Not serious | Not available | MD | Off-label use | Acute myeloid leukemia refractory |
| Spontaneous | 538271 | 14/03/2019 | 60 | M | Not serious | Not available | MD | Off-label use | Acute myeloid leukemia relapsed |
| Spontaneous | 538272 | / | 79 | F | Not serious | Not available | MD | Off-label use | Acute myeloid leukemia refractory |
| Spontaneous | 538288 | 17/03/2019 | 79 | F | Serious—hospitalization | Not yet resolved | MD | Increased creatinine, Increased LDH, Hyperpyrexia, Hyperferritinemia | Non-Hodgkin's lymphoma |
| Spontaneous | 538544 | 01/03/2019 | 70 | F | Not serious | Not available | MD | Off-label use | Acute myeloid leukemia relapsed |
| Spontaneous | 538546 | / | 70 | F | Serious—hospitalization | Not available | MD | hypertensive crisis | Chronic lymphocytic leukemia |
| Spontaneous | 540345 | 01/01/2019 | / | F | Not serious | Not available | MD | Off-label use | Acute myeloid leukemia |
| Spontaneous | 540802 | 01/01/2019 | 35 | M | Serious—other clinically relevant condition | Not yet resolved | MD | Disease progression | Acute myeloid leukemia |
| Spontaneous | 540804 | 11/03/2019 | / | M | Serious—other clinically relevant condition | Fully recovered | MD | Off-label use, disease progression | Acute myeloid leukemia |
| Spontaneous | 540986 | / | 50 | M | Not serious | Not yet resolved | MD | Off-label use | Acute myeloid leukemia |
| Spontaneous | 540987 | 19/03/2019 | / | F | Not serious | Not available | MD | Off-label use | Acute myeloid leukemia |
| Spontaneous | 541263 | 01/03/2019 | / | M | Not serious | Not available | MD | Off-label use | Multiple myeloma |
| Spontaneous | 541382 | / | 78 | M | Serious—other clinically relevant condition | Not available | MD | Disease progression | Acute myeloid leukemia |
| Spontaneous | 541384 | 30/03/2019 | / | M | Not serious | Not available | MD | Off-label use | Acute myeloid leukemia |
| Spontaneous | 541538 | 01/01/2019 | / | M | Not serious | Not available | MD | Off-label use | Acute myeloid leukemia |
| Spontaneous | 541539 | 01/03/2019 | / | M | Not serious | Not available | MD | Off-label use | Acute myeloid leukemia |
| Spontaneous | 542404 | / | / | M | Serious-death | Death | MD | Off-label use, disease progression | Acute myeloid leukemia relapsed |
| Spontaneous | 542801 | 01/03/2019 | / | F | Not serious | Not available | MD | Off-label use | Acute myeloid leukemia |
| Spontaneous | 543082 | / | 67 | M | Serious—other clinically relevant condition | Not available | MD | Off-label use | Acute myeloid leukemia relapsed |
| Spontaneous | 543083 | 05/03/2019 | / | M | Not serious | Not available | MD | Off-label use | Acute myeloid leukemia |
| Spontaneous | 543526 | / | 33 | F | Serious—other clinically relevant condition | Fully recovered | MD | Disease progression, febrile neutropenia | Acute myeloid leukemia refractory |
| Spontaneous | 543608 | / | 73 | M | Not serious | Not available | MD | Off-label use | Acute myeloid leukemia |
| Spontaneous | 544958 | / | 70 | M | Serious—other clinically relevant condition | Not available | MD | Intrahepatic cholangiocarcinoma | Chronic lymphocytic leukemia |
| Spontaneous | 544959 | / | / | M | Not serious | Not yet resolved | Patient | Edema, Weight gain | / |
| From non-intervantional study | 545069 | / | 59 | / | Serious-death | Death | MD | Septic Shock | Chronic lymphocytic leukemia refractory |
| Spontaneous | 545070 | 01/10/2018 | / | F | Not serious | Not available | MD | Off-label use | Acute myeloid leukemia |
| Spontaneous | 545071 | 09/04/2019 | / | M | Not serious | Not available | MD | Off-label use | Acute myeloid leukemia |
| Spontaneous | 545377 | 15/04/2019 | / | M | Not serious | Not yet resolved | MD | Off-label use | Acute myeloid leukemia |
| Spontaneous | 548228 | / | 68 | F | Not serious | Not available | MD | Off-label use | Acute myeloid leukemia refractory |
| Spontaneous | 548313 | 01/04/2019 | / | M | Not serious | Not available | MD | Off-label use | Waldenstrom macroglobulinemia |
| Spontaneous | 548871 | / | 58 | F | Not serious | Not available | MD | Off-label use | Acute myeloid leukemia refractory |
| Spontaneous | 548873 | 03/05/2019 | / | M | Not serious | Not available | MD | Off-label use | Acute myeloid leukemia |
| Spontaneous | 549559 | 11/01/2019 | 75 | F | Serious—other clinically relevant condition | Improved | Pharmacist | Neutropenia | Chronic lymphocytic leukemia |
| Spontaneous | 549561 | 01/02/2019 | 75 | F | Serious—other clinically relevant condition | Not available | Pharmacist | Thrombocytopenia | Chronic lymphocytic leukemia |
| Spontaneous | 549926 | / | 65 | M | Not serious | Not available | MD | Urticaria | Chronic lymphocytic leukemia |
| Spontaneous | 549982 | 30/04/2019 | 65 | M | Not serious | Not yet resolved | MD | Macular rash | lymphocytic leukemia |
| Spontaneous | 551565 | 01/04/2019 | / | M | Not serious | Fully recovered | MD | Off-label use | Acute myeloid leukemia |
| Spontaneous | 551567 | 01/05/2019 | / | M | Not serious | Not available | MD | Off-label use | Acute myeloid leukemia |
| Spontaneous | 551840 | 01/03/2019 | / | M | Not serious | Not available | MD | Off-label use | Acute myeloid leukemia |
| Spontaneous | 551992 | / | / | M | Not available | Not available | Pharmacist | constipation | / |
| Spontaneous | 553191 | / | 71 | M | Serious—other clinically relevant condition | Not available | MD | Disease progression | Acute myeloid leukemia relapsed |
| Spontaneous | 553192 | / | 57 | M | Not serious | Not available | MD | Off-label use | Acute myeloid leukemia refractory |
| Spontaneous | 553193 | / | 67 | M | Not serious | Not available | MD | Off-label use | Acute myeloid leukemia relapsed |
| Spontaneous | 553194 | 01/04/2019 | / | F | Not serious | Not available | MD | Off-label use | Acute myeloid leukemia |
| Spontaneous | 553491 | 01/03/2019 | / | M | Not serious | Not available | MD | Off-label use | Acute myeloid leukemia |
| Spontaneous | 553493 | / | 71 | M | Not serious | Not available | MD | Off-label use | Acute myeloid leukemia refractory |
| Spontaneous | 554860 | 01/04/2019 | / | F | Not serious | Not available | MD | Off-label use | Acute myeloid leukemia |
| Spontaneous | 554861 | 01/04/2019 | / | F | Not serious | Not available | MD | Off-label use | Acute myeloid leukemia |
| Spontaneous | 555113 | 01/04/2019 | / | M | Not serious | Fully recovered | MD | Off-label use, Erythema | Acute myeloid leukemia |
| Spontaneous | 555114 | 01/04/2019 | / | F | Not serious | Not available | Healthcare professional (other) | Off-label use | Acute myeloid leukemia |
| Spontaneous | 555187 | 01/04/2019 | / | F | Not serious | Not available | MD | Off-label use | Acute myeloid leukemia |
| Spontaneous | 556043 | 01/08/2018 | / | M | Not serious | Not available | MD | Off-label use | Acute myeloid leukemia |
| Spontaneous | 556045 | 01/06/2019 | / | M | Not serious | Not available | MD | Off-label use, infection | Acute myeloid leukemia |
| Spontaneous | 556367 | 01/01/2019 | / | M | Not serious | Not available | MD | Off-label use | Acute myeloid leukemia |
| Spontaneous | 556495 | 20/05/2019 | 62 | M | Serious—other clinically relevant condition | Not yet resolved | MD | Pulmonary aspergillosis | Chronic leukemia |
| Spontaneous | 556626 | 01/11/2018 | / | M | Not serious | Not available | MD | Off-label use | Acute myeloid leukemia |
| Spontaneous | 556627 | 01/11/2018 | / | F | Not serious | Not available | MD | Off-label use | Acute myeloid leukemia |
| Spontaneous | 556677 | / | 71 | M | Not serious | Not available | MD | Off-label use | Acute myeloid leukemia |
| Spontaneous | 556678 | / | / | F | Not serious | Not available | MD | Off-label use, Hyperpyrexia | Acute myeloid leukemia |
| Spontaneous | 556845 | / | 61 | M | Not serious | Not available | MD | Pulmonary aspergillosis | Chronic lymphocytic leukemia |
| Spontaneous | 556914 | 01/12/2018 | / | F | Not serious | Not available | MD | Off-label use | Acute myeloid leukemia |
| Spontaneous | 556915 | 01/01/2019 | / | M | Not serious | Not available | MD | Off-label use | Acute myeloid leukemia |
| Spontaneous | 557232 | 01/01/2019 | / | M | Not serious | Fully recovered | MD | Off-label use, Cytopenia | Blastic plasmacytoid dendritic cell neoplasm |
| Spontaneous | 557311 | 01/05/2019 | / | M | Not serious | Not available | MD | Off-label use | Acute myeloid leukemia |
| Spontaneous | 557627 | 01/05/2018 | / | M | Not serious | Not available | MD | Off-label use | Acute myeloid leukemia |
| Spontaneous | 557628 | / | / | M | Serious—other clinically relevant condition | Fully recovered | MD | Off-label use | Diffuse large B-cell lymphoma |
| Spontaneous | 560446 | 17/04/2019 | / | M | Not serious | Not available | MD | Off-label use | Acute myeloid leukemia |
| Spontaneous | 560447 | 01/01/2019 | / | M | Serious—other clinically relevant condition | Fully recovered | MD | Off-label use, cerebral hemorrhage | Acute myeloid leukemia |
| Spontaneous | 561371 | / | 50 | M | Not serious | Not available | MD | Off-label use | Acute myeloid leukemia |
| Spontaneous | 562013 | / | / | M | Not serious | Not available | MD | Off-label use | Acute myeloid leukemia refractory |
| Spontaneous | 562014 | / | 80 | F | Not serious | Not available | MD | Off-label use | Acute myeloid leukemia refractory |
| Spontaneous | 562015 | / | 76 | F | Not serious | Not available | MD | Off-label use | Acute myeloid leukemia refractory |
| Spontaneous | 562117 | / | 70 | F | Not serious | Not available | MD | Off-label use | Acute myeloid leukemia refractory |
| Spontaneous | 562752 | / | 77 | M | Not serious | Not available | MD | Off-label use | Acute myeloid leukemia |
| Spontaneous | 564935 | 01/05/2019 | / | F | Serious—other clinically relevant condition | Not yet resolved | MD | Off-label use | Acute myeloid leukemia |
| Spontaneous | 564936 | / | 72 | M | Serious—other clinically relevant condition | Not available | MD | Off-label use | Acute myeloid leukemia |
| Spontaneous | 565481 | / | 71 | M | Serious—other clinically relevant condition | Not yet resolved | MD | Off-label use | Acute myeloid leukemia |
| Spontaneous | 566025 | 01/06/2019 | / | M | Not serious | Not available | MD | Off-label use | Acute myeloid leukemia |
| Spontaneous | 566026 | 01/05/2019 | / | M | Not serious | Not available | MD | Off-label use | Acute myeloid leukemia |
| Spontaneous | 566143 | 02/07/2019 | 50 | M | Serious—hospitalization | Not yet resolved | MD | Epidermolysis bullosa | Acute myeloid leukemia relapsed |
| Spontaneous | 567563 | 08/07/2019 | 50 | M | Serious—hospitalization | Not yet resolved | MD | Off-label use, Erythroderma-like rash | Acute myeloid leukemia refractory |
| Spontaneous | 568866 | 01/07/2019 | 63 | M | Not serious | Not available | MD | Nausea | / |
| Spontaneous | 572533 | 17/08/2019 | 44 | M | Serious—hospitalization | Fully recovered | Healthcare professional (other) | Fever, Skin rash | Chronic lymphocytic leukemia |
| Spontaneous | 572535 | 19/08/2019 | / | F | Not serious | Not available | MD | Off-label use | Acute myeloid leukemia |
| Spontaneous | 573246 | / | 56 | M | Not serious | Not available | MD | Off-label use | Acute myeloid leukemia refractory |
| Spontaneous | 573487 | / | 71 | M | Not serious | Not available | MD | Off-label use | Acute myeloid leukemia refractory |
| Spontaneous | 576046 | 01/08/2019 | / | M | Not serious | Not available | MD | Off-label use | Acute myeloid leukemia |
| From non-intervantional study | 577062 | 01/01/2019 | 63 | M | Serious-death | Death | MD | Drug interaction, Supraventricular paroxysmal tachycardia | Mantle cell lymphoma |
| Spontaneous | 577110 | / | 74 | F | Not serious | Not available | MD | Off-label use | Acute myeloid leukemia |
| Spontaneous | 578109 | 09/09/2019 | / | M | Not serious | Not available | MD | Off-label use | Acute myeloid leukemia |
| Spontaneous | 579333 | 30/09/2019 | / | M | Serious-death | Death | MD | Off-label use, infection | Acute myeloid leukemia |
| Spontaneous | 580515 | / | 55 | F | Not serious | Not available | MD | Off-label use | Acute myeloid leukemia |
| Spontaneous | 580797 | 20/05/2019 | 62 | M | Not serious | Fully recovered | MD | Pneumonia | Chronic leukemia |
| Spontaneous | 583212 | 01/09/2019 | / | M | Not serious | Not available | MD | Off-label use | Acute myeloid leukemia |
| Spontaneous | 583431 | / | 42 | F | Serious—other clinically relevant condition | Not available | MD | Disease progression | Acute myeloid leukemia refractory |
| Spontaneous | 583584 | / | 75 | F | Serious—other clinically relevant condition | Not available | MD | Off-label use | Acute myeloid leukemia refractory |
| Spontaneous | 583585 | 01/08/2019 | 47 | F | Not serious | Not available | MD | Off-label use | Mantle cell lymphoma |
| Spontaneous | 583872 | 01/09/2019 | / | M | Not serious | Not available | MD | Off-label use | Acute myeloid leukemia |
| Spontaneous | 584885 | 01/05/2019 | / | M | Not serious | Not available | MD | Off-label use | Acute myeloid leukemia |
| Spontaneous | 585892 | / | 69 | M | Not serious | Not available | MD | Off-label use | Acute myeloid leukemia refractory |
| Spontaneous | 585893 | / | 40 | F | Not serious | Not available | MD | Off-label use | Acute myeloid leukemia refractory |
| Spontaneous | 586030 | 06/11/2019 | 75 | M | Serious—other clinically relevant condition | Not yet resolved | Pharmacist | Neutropenia | Chronic lymphocytic leukemia |
| Spontaneous | 586108 | 01/08/2019 | / | F | Not serious | Not available | MD | Off-label use | Acute myeloid leukemia |
| Spontaneous | 587865 | 28/10/2019 | / | M | Not serious | Not available | MD | Off-label use | Acute myeloid leukemia |
| Spontaneous | 588072 | 28/10/2019 | 75 | F | Not serious | Improved | MD | Increased transaminase | Leukemia |
| Spontaneous | 588995 | 14/11/2019 | 77 | M | Not serious | Not available | Pharmacist | Diarrhea | / |
| Spontaneous | 589231 | 04/11/2019 | / | F | Not serious | Not available | MD | Off-label use | Acute myeloid leukemia |
| Spontaneous | 589684 | 01/01/2019 | / | M | Serious—other clinically relevant condition | Fully recovered | MD | Increased Lymphocyte Absolute Count, Kidney Failure, Splenomegaly, tumor Lysis Syndrome | Chronic lymphocytic leukemia |
| Spontaneous | 590132 | 01/11/2019 | / | M | Not serious | Not available | MD | Off-label use | Acute myeloid leukemia |
| Spontaneous | 590771 | / | / | / | Serious—other clinically relevant condition | Not available | MD | Central nervous system disease | Acute myeloid leukemia |
| Spontaneous | 590772 | 01/09/2019 | 62 | F | Not serious | Not available | MD | Off-label use | Acute myeloid leukemia |
| Spontaneous | 590989 | / | 78 | F | Not serious | Not available | MD | Off-label use | Acute myeloid leukemia refractory |
| Spontaneous | 590990 | / | 82 | M | Not serious | Not available | MD | Off-label use | Acute myeloid leukemia refractory |
| Spontaneous | 590991 | / | 68 | M | Not serious | Not available | MD | Off-label use | Acute myeloid leukemia refractory |
| Spontaneous | 593667 | 16/05/2019 | 68 | M | Serious—other clinically relevant condition | Fully recovered | MD | Interstitial pneumonia, Fever | Acute myeloid leukemia |
| Spontaneous | 593700 | 01/11/2019 | 59 | M | Not serious | Not available | MD | Fever, Skin rash | Mantle cell lymphoma |
| Spontaneous | 593904 | 03/12/2019 | / | F | Not serious | Not available | MD | Off-label use | Acute myeloid leukemia |
| Spontaneous | 594184 | 01/11/2019 | / | M | Not serious | Not available | MD | Off-label use | Mantle cell lymphoma |
| Spontaneous | 599148 | 17/12/2019 | 72 | M | Serious—hospitalization | Fully recovered | Healthcare professional (other) | Sepsis | Chronic lymphocytic leukemia |
| Spontaneous | 604144 | 15/11/2019 | 65 | F | Serious—other clinically relevant condition | Not available | MD | Febrile Neutropenia | Chronic lymphocytic leukemia |
| Spontaneous | 604606 | 22/11/2019 | 72 | F | Serious—other clinically relevant condition | Fully recovered | Pharmacist | Neutropenia | Chronic lymphocytic leukemia |
| Spontaneous | 605264 | 29/01/2020 | 69 | M | Not serious | Not yet resolved | MD | Diffuse rash | Chronic lymphocytic leukemia |
| Spontaneous | 611769 | 15/01/2020 | 79 | M | Not serious | Not available | MD | Itching | Chronic lymphocytic leukemia |
| From study (individual use - compassionate use, named patient basis) | 615770 | 08/04/2020 | 57 | M | Serious-death | Not related to the drug | MD | Dyspnea, Fever, Disease progression | Acute lymphocytic leukemia |
| Spontaneous | 616993 | 27/08/2019 | 79 | F | Serious—other clinically relevant condition | Fully recovered | Pharmacist | Off label use, diarrhea | Acute myeloid leukemia |
| Spontaneous | 620349 | 02/06/2019 | 61 | M | Serious—hospitalization | Improved | MD | Leukopenia, Pancytopenia, thrombocytopenia | Myeloid leukemia |
| Spontaneous | 622072 | 20/02/2020 | 63 | F | Serious—other clinically relevant condition | Not yet resolved | Pharmacist | Pancytopenia | Acute myeloid leukemia |
| From study (individual use - compassionate use, named patient basis) | 628630 | 14/07/2020 | 61 | M | Serious—hospitalization | Fully recovered | MD | Tumor lysis syndrome | Mantle cell lymphoma |
| From non-intervantional study | 630889 | 10/08/2020 | 75 | / | Serious—hospitalization | Not yet resolved | MD | Asthenia, Hyperbilirubinemia, International normalized ratio increased | Acute myeloid leukemia |

**Supplementary table 2:** type of adverse drug reaction (ADR) related to the use of venetoclax within the Italian spontaneous ADR reporting database (Italian National Network of Pharmacovigilance, Rete Nazionale di Farmacovigilanza, RNF)

| **ADR** | **N** | **%** |
| --- | --- | --- |
| Therapeutic response (lack/loss) | 32 | 12,2 |
| Hematological | 11 | 4,2 |
| Infection | 15 | 5,7 |
| Off-label | 168 | 63,9 |
| Cutaneous | 11 | 4,2 |
| GI | 6 | 2,3 |
| Other | 20 | 7,6 |

**Supplementary table 3:** seriousness and outcome of adverse drug reactions reported in Italy (excluding those reporting off-label use only).

| **Seriousness** | **n** | **%** |
| --- | --- | --- |
| Not available | 1 | 1,1 |
| Not serious | 26 | 27,4 |
| Serious—death | 22 | 23,2 |
| Serious—hospitalization | 15 | 15,8 |
| Serious—other clinically relevant condition | 31 | 32,6 |
| **Outcome** | **n** | **%** |
| Death | 21 | 22,1 |
| Improved | 23 | 24,2 |
| Not reported | 6 | 6,3 |
| Not yet resolved | 33 | 34,7 |
| Fully recovered | 1 | 1,1 |
| Death-Not related to the drug | 11 | 11,6 |
